# Neural Processing of Spectral and Durational Changes in Speech and Non-speech Stimuli: An MMN Study With Czech Adults

**DOI:** 10.3389/fnhum.2021.643655

**Published:** 2021-08-09

**Authors:** Natalia Nudga, Josef Urbanec, Zuzana Oceláková, Jan Kremláček, Kateřina Chládková

**Affiliations:** ^1^Faculty of Arts, Institute of Phonetics, Charles University, Prague, Czechia; ^2^Department of Pathological Physiology, Faculty of Medicine in Hradec Králové, Charles University, Hradec Králové, Czechia; ^3^Pediatrics Department, Havlíčkův Brod Hospital, Havlíčkův Brod, Czechia; ^4^Department of Medical Biophysics, Faculty of Medicine in Hradec Králové, Charles University, Hradec Králové, Czechia; ^5^Faculty of Arts, Institute of Czech Language and Theory of Communication, Charles University, Prague, Czechia; ^6^Institute of Psychology, Czech Academy of Sciences, Brno, Czechia

**Keywords:** mismatch negativity, auditory processing, vowels, phonology, perceptual asymmetries

## Abstract

Neural discrimination of auditory contrasts is usually studied via the mismatch negativity (MMN) component of the event-related potentials (ERPs). In the processing of speech contrasts, the magnitude of MMN is determined by both the acoustic as well as the phonological distance between stimuli. Also, the MMN can be modulated by the order in which the stimuli are presented, thus indexing perceptual asymmetries in speech sound processing. Here we assessed the MMN elicited by two types of phonological contrasts, namely vowel quality and vowel length, assuming that both will elicit a comparably strong MMN as both are phonemic in the listeners’ native language (Czech) and perceptually salient. Furthermore, we tested whether these phonemic contrasts are processed asymmetrically, and whether the asymmetries are acoustically or linguistically conditioned. The MMN elicited by the spectral change between /a/ and /ε/ was comparable to the MMN elicited by the durational change between /ε/ and /ε:/, suggesting that both types of contrasts are perceptually important for Czech listeners. The spectral change in vowels yielded an asymmetrical pattern manifested by a larger MMN response to the change from /ε/ to /a/ than from /a/ to /ε/. The lack of such an asymmetry in the MMN to the same spectral change in comparable non-speech stimuli spoke against an acoustically-based explanation, indicating that it may instead have been the phonological properties of the vowels that triggered the asymmetry. The potential phonological origins of the asymmetry are discussed within the featurally underspecified lexicon (FUL) framework, and conclusions are drawn about the perceptual relevance of the place and height features for the Czech /ε/-/a/ contrast.

## Introduction

Speech perception is a cognitive process which transforms the acoustic signal into respective neural representations in the human brain. One of the most fundamental properties of human speech perception is the ability to detect phonetic and phonological contrasts. Sensitivity to such contrasts has been examined by the means of behavioral tests (discrimination or categorization tasks) (Repp and Crowder, 1990; Polka and Bohn, 2003; Johnson, 2015) as well as via techniques that monitor brain activity, such as event-related potentials (ERPs) measured with electroencephalography (EEG; Eulitz and Lahiri, 2004; De Jonge and Boersma, 2015) or their magnetic equivalents measured with magnetoencephalography (Scharinger et al., 2016; Højlund et al., 2019). The most common ERP component used to study the brain response to an auditory contrast is the mismatch negativity (MMN). The MMN response is elicited by an irregularity, typically when a series of frequently presented stimuli, standards, is interrupted by a different infrequent stimulus, deviant. ERP studies show that the magnitude of the MMN reflects the extent of the perceived difference between the standard and deviant, whereby not only the acoustic distance but also the category membership of the stimuli modulate the strength of the response (Näätänen et al., 1997). The MMN can thus be used to estimate the linguistic importance and relevance of phonetic differences between stimuli for speech perception.

The auditory ERP component MMN and its magnetic correlate MMNm have been used to assess the neural processing of both vowels and consonants, and to study the relevance of qualitative, or less commonly, quantitative phonemic contrasts. Ylinen et al. (2005) studied the processing of consonant quality and quantity via MMN, focusing on stop consonants /p/, /p:/, /t/, and /t:/. In their experiment, the plosive [t:] served as the standard, [t] as a quantity deviant, [p:] as a quality deviant, and [p] as a double deviant (all embedded in the same [i_i] frame). The MMN elicited by the double deviant was approximately equal to the sum of the quantity- and quality-deviant MMNs and the authors concluded that consonant quality and quantity are processed independently. Their results also show that the quantitative change of the consonant elicited greater and earlier MMN response than the qualitative change. This finding of differential strength of processing of phoneme quality and quantity could be specific to plosive consonants. In vowels, for instance, a change in quality is much more salient than a change in plosive consonant place of articulation. The question thus remains how robustly quality versus quantity changes are processed in vowels.

Previous studies focusing on vowels show that changes in vowel spectral quality elicit a larger MMN in listeners for whom these changes represent a linguistic, i.e., phonemic change, than in listeners for whom these changes are not phonemic (Näätänen et al., 1997). Similarly, changes in the duration of vowels elicit a stronger MMN response in listeners in whose native language vowel length is phonemic than in listeners for whom it is not (Kirmse et al., 2008; Hisagi et al., 2010; Chládková et al., 2013). The effect of native phoneme inventory on both vowel quality and vowel length processing is indisputable, however, it has not yet been shown how the neural processing of vowel length and vowel spectral quality compare to one another. The present study therefore aims to investigate and compare the neural processing of vowel duration and vowel quality of adult speakers of a language in which both vowel quality and vowel length have a contrastive role. Obtained results will also show if MMNs evoked by changes in vowel quality and quantity match with the pattern obtained by Ylinen et al. (2005) for plosive consonants, in which greater average MMN was observed in case of a quantity change.

A number of studies exploring the sensitivity to phonemic contrasts have encountered a phenomenon called perceptual asymmetry. Perceptual asymmetries can be observed when participants more readily process or respond to a change when category A is presented before category B than vice versa. Such findings imply that the perceptual space differs from the physical space and that due to its asymmetric nature its properties cannot be captured by Euclidean geometry (e.g., distances in the vowel formant space). Asymmetry in perception has been investigated for various types of stimuli including color, line orientation, numbers (Rosch, 1975), geometric figures (Tversky and Gati, 1978) as well as vowels (Polka and Bohn, 2003, 2011; Eulitz and Lahiri, 2004; De Jonge and Boersma, 2015), and consonants (Schluter et al., 2016; Cummings et al., 2017; Højlund et al., 2019). Vowel perception asymmetry has been studied by means of reaction time or accuracy in discrimination tasks, where a reversed order of stimuli led to the significant difference in the measured parameters. Asymmetrical perception of vowels has also been attested in neurolinguistic MMN studies, when the roles of standard and deviant stimuli were switched (Eulitz and Lahiri, 2004; De Jonge and Boersma, 2015). For instance, De Jonge and Boersma (2015) found asymmetrical patterns in vowel perception when comparing MMN responses of French listeners to contrasts among four French vowels [y, u, ø, o]. Their results showed that the MMN evoked by a change from a high vowel such as [u] toward a high-mid vowel such as [o], and by a change from a back vowel such as [u] to a front vowel such as [y] was significantly larger (i.e., more negative) than vice versa. In addition to the asymmetry, they found that the average MMN resulting from a change in vowel place (backness or frontness) was significantly larger compared to the MMN resulting from a change in vowel height.

There are several hypotheses and theories that offer explanation to the perceptual asymmetry phenomena. According to Repp and Crowder (1990), perceptual asymmetries are caused by different rates of memory decay, which, as the authors argued, is slower for more prototypical (or less ambiguous) vowels. They concluded that at either point of a vowel continuum the difference between stimuli is more detectable when the more salient vowel comes second in a pair, and thus serves as the subject of comparison.

Polka and Bohn (2003, 2011) proposed the natural referent vowel (NRV) framework which operates with the concept of peripheral vowels and aims to explain language-general, i.e., auditorily-based, patterns in infant speech perception. Peripherality acoustically coincides with formant focalization, that is the convergence of two formant frequencies in a vowel (Schwartz et al., 2005). In a focal vowel, the proximity of two formants strengthens their respective amplitudes and results in a perceptually prominent frequency band. According to the NRV framework, a difference is more detectable for a change from a less peripheral, or non-focal, to a more peripheral, or focal, vowel than vice versa. Along those lines, the difference between two vowels such as [u] and [y] should be more readily detectable, i.e., perceived as greater, when [y] is presented before [u] than vice versa. Note that such NRV-based asymmetry is opposite to the asymmetries obtained by De Jonge and Boersma (2015) who tested adults (and it is opposite also to the asymmetries obtained by Wanrooij et al., 2014 for infants). Although not originally proposed as an explanation for asymmetries in the *neural* processing of vowels, it seems viable that a more detectable difference between stimuli leads to a stronger MMN response (as shown by e.g., Näätänen et al., 1997). Therefore, the NRV can be used to formulate acoustically-based predictions for MMN such that a focal (i.e., perceptually more salient) deviant should elicit a stronger MMN than a non-focal deviant.

Repp and Crowder as well as Polka and Bohn have based their theories of vowel perception asymmetry on the acoustic properties of vowels, while other authors, namely, Lahiri and Reetz (2002) have approached this phenomenon from the phonological point of view and formulated the featurally underspecified lexicon (FUL) theory. Their theory explains the perceptual asymmetries through reference to phonological representations, postulating that a change from a stimulus specified for a particular phonological feature to a stimulus underspecified for that feature is processed more strongly than a change in the reversed order. The predictions of the FUL theory have been borne out by a number of studies (Eulitz and Lahiri, 2004; Lipski et al., 2007; Scharinger et al., 2012, 2016; De Jonge and Boersma, 2015; Schluter et al., 2016).

Considering a vowel contrast such as one between a focal and phonologically specified /a/ and a non-focal and underspecified /ε/, one can see that an NRV-like asymmetry predicted by acoustics (i.e., a stronger response to a change from /ε/ to /a/) does not necessarily coincide with an asymmetry predicted by the phonological FUL framework (i.e., a stronger response to a change from /a/ to /ε/). Crucially, predictions based on phonological representations can also differ depending on the adopted phonological theory. If we again consider the vowels /a/ and /ε/, then according to the FUL theory, /ε/ is underspecified for feature [LOW]. However, in Element theory (Harris and Lindsey, 1995) which describes vowels in terms of elements | A|, | I|, and | U|, it is /a/ that contains 1 element and is thus underspecified in comparison to /ε/ which contains 2 elements. Consequently, one could hypothesize that it is /a/ and not /ε/ that should evoke greater MMN response when presented as a deviant. Although the predicted perceptual (MMN) asymmetries differ across phonological frameworks, they have been mainly tested within the FUL framework. An exception is De Jonge and Boersma (2015) who contrasted FUL and Element theory and whose MMN data from French adults supported FUL. Because it is the most widely researched phonological framework in the MMN literature, the present study adopts FUL as the basis for phonological predictions and contrasts it with NRV-like acoustic predictions.

As introduced above, the present experiment focuses on the MMN to vowel quality and vowel length contrasts which are both phonemic in the listeners’ native language, Czech. The specific contrasts are /ε/-/a/ and /ε/-/ε:/, for vowel quality and vowel length, respectively. Since spectrum can be a secondary perceptual cue to vowel length, we have selected the /ε/-/ε:/ pair out of the five short-long pairs in Czech because it entails the smallest spectral difference, both in perception (Podlipský et al., 2019) and production (Paillereau and Chládková, 2019). Besides comparing the strength of the MMN elicited by the two distinct types of phonemic changes, the present experiment tests whether any MMN asymmetries exist for those vowel contrasts and if yes, whether they are phonologically or acoustically motivated.

In order to provide a further test of whether any potential asymmetries are more likely attributable to the phonology or to the acoustics, we compare Czech listeners’ processing of the two vowel contrasts /ε/-/a/ and /ε/-/ε:/ to their processing of identical acoustic differences in non-speech stimuli. The non-speech stimuli are inharmonic tone complexes with the first three formant frequencies and duration identical to those of the vowels /a/, /ε/, and /ε:/; they are thus comparably complex as the vowels but not confusable with speech. If the potential asymmetries are acoustically conditioned, they should be found in both the non-speech and the speech conditions in the present study. If, on the contrary, the asymmetries are (at least to some extent) phonologically based the pattern of results should differ across speech and non-speech.

According to Polka and Bohn (2003, 2011), the acoustic properties of our stimuli predict a greater MMN when a focal vowel (or tone complex) is the deviant and a non-focal vowel (or tone complex) is the standard. In that respect, the vowel /a/ and the /a/-like tone are focal because their first and second formants are close to one another, concentrating (focalized) energy in the F1–F2 frequency band. In contrast, the first and second formants of the vowel /ε/ and the /ε/-like tone are relatively far apart and thus contain non-focalized energy. Acoustically, the change from the non-focal /ε/ (-like tone) to the focal /a/ (-like tone) should elicit a stronger MMN response than a reverse change. As for the durational dimension, for which focalization has not been formally defined, intuitively a longer stimulus is more prominent than a shorter stimulus. The acoustically-motivated prediction then is that a change from the short /ε/ (-like tone) to the long /ε/ (-like tone) will elicit a greater MMN than vice versa. This direction of predicted asymmetry is further in line with previous findings that the addition of information is more detectable than its deletion (Timm et al., 2011).

The alternative, phonologically-based predictions for asymmetries are made in accordance with the featural (under)specification framework (Lahiri and Reetz, 2010), which states that the magnitude of the MMN will be greater in case of change from a fully specified vowel to an underspecified vowel than vice versa. Czech central low vowel /a/ and front mid vowel /ε/ differ both in the horizontal plane and in height, nevertheless from the phonological point of view there are distinguished only by means of the feature [LOW] (which is specified for /a/ but not for /ε/) as they are both underspecified with respect to the feature [BACK]. Therefore, in conformity with the FUL theory, we expect a greater MMN response when underspecified /ε/ is a deviant. Regarding the quantity contrast, according to some authors the difference between Czech /ε/ and /ε:/ lies in the feature [LONG], which is specified for /ε:/ (Palková, 1994, p. 206, Skarnitzl et al., 2016, p. 101). This means that in the vowel quantity condition, /ε/ is again underspecified, and the MMN should be larger when /ε/ is a deviant and /ε:/ is a standard.

Predictions of the vowel perception asymmetry in terms of relative magnitude of the MMN response are summarized in Table 1. For the complex tone stimuli, the asymmetrical behavior is expected based solely on the acoustical approach, and thus coincides with the first row of Table 1.

**TABLE 1:**
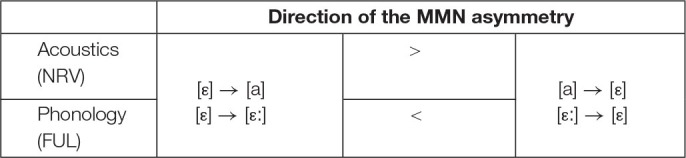
Acoustically- and phonologically-based predictions of relative magnitude of the MMN response to the experimental stimuli.

To sum up, the present study has two goals. Firstly, it compares the neural processing of vowel length and vowel quality in a language that uses both types of contrasts phonemically [similarly to the comparison of consonantal quality and consonantal length reported by Ylinen et al. (2005)]. Secondly, it tests whether there are any directional asymmetries in the perception of vowel length and/or vowel quality and whether they can be explained by the vowels’ acoustic properties or phonological specification.

## Materials and Methods

### Stimuli

We created two sets of stimuli, one set for the speech condition and one set for the non-speech condition. The speech stimuli were naturally produced, edited consonant-vowel (CV) syllables [fε] and [fa]. The formants were stable throughout the vowels and corresponded to the Czech low-mid front /ε/ and low /a/, respectively. The first three formants of [ε] in [fε] were 755 Hz, 1646 Hz, and 2710 Hz, and the first three formants of [a] in [fa] were 864, 1287, and 2831 Hz; these values are in line with the formants of Czech vowels produced by women reported by Skarnitzl and Volín (2012). The duration of the vowels [ε] and [a] (extracted from the CV frames) was modified using PSOLA in Praat (Boersma and Weenink, 1992–2020). The vowel [a] had a duration of 220 ms, and [ε] was resynthesized with three durations, namely, 220, 180, and 360 ms, which met the following conditions: 220 ms was judged (by three expert phoneticians) as a typical duration of the mid and low short vowels in an isolated CV syllable, 360 ms represented a long vowel in a CV syllable that was not perceived as unnaturally exaggerated, and short /ε/ with the duration of 180 ms was considered to be sufficiently distinct from the long /ε:/.^1^ In order to create the stimuli, we cut out the initial fricative consonant [f] from one recorded syllable and combined it with the target [a] and [ε] vowels, such that the fricative [f] was identical across all four speech stimuli and had a duration of 150 ms. None of the created [f] + V syllables carries lexical or morphological content in Czech. The speech stimuli had been used in a behavioral study on vowel perception with Czech-exposed infants (Paillereau et al., 2021), and recently, along with the non-speech stimuli described below, in an ERP study with Czech newborns (Chládková et al., under review).

To test the discrimination of a spectral contrast, the non-focal [fε] and the focal [fa] lasting for 220 ms each were used. The vowel [a] is considered focal because the distance between its first and second formant is *d*_*a*_ = 2.07 Bark, while the vowel [ε] in [fε] is non-focal because its first two formants are spread apart by *d*_ε_ = 4.08 Bark. The difference between [a] and [ε] thus lies in their perceptual prominence, where [a] is the more prominent one. The discrimination of a durational contrast was tested by the short 180-ms [fε] and long 360-ms [fε]. Similarly as for the spectral dimension, the short and the long vowel differ in their perceptual prominence, where the short one contains energy over a shorter time interval (i.e., less energy in total) as can thus be seen as perceptually less prominent stimulus than a long vowel represented by energy in a longer time interval. The intensity of the stimuli was scaled by peak to be matched across all the 4 different syllables.

The non-speech stimuli were inharmonic tone complexes with spectral and durational properties mimicking those of the vowels described above. Inharmonic tone complexes are comparably complex as vowels in that their source signal contains a series of fundamental frequency harmonics and is filtered with vocal-tract like formants. At the same time, the inharmonic tone complexes are not confusable with vowels because their source signal frequencies are spaced inharmonically (Goudbeek et al., 2009; Scharinger et al., 2014). The tone complexes in the present experiment had 15 inharmonically spaced frequency components, the first one at 500 Hz and every following being 1.15 times higher. The inharmonic source signal was filtered with three formants, namely, for the focal spectral condition with the formants of [a], for the non-focal spectral condition and the short and long durational condition with the formants of [ε]. Durations of the non-speech stimuli were identical to the durations of the vowels from the speech condition. The amplitude was ramped linearly over 5 ms at stimulus onset and offset. Sound intensity was scaled to be identical across all the four stimuli. As in the speech condition, the [a]-like focal tone (prominent) and the [ε]-like non-focal 220-ms tone (non-prominent) were used to test discrimination of spectral differences, and the 180-ms [ε]-like tone (non-prominent) and the 360-ms [ε]- like tone (prominent) were used to test discrimination of duration differences.

### Presentation Paradigm

The stimuli, i.e., the individual syllables or the individual tone complexes, were presented in a roving-standard paradigm (Haenschel et al., 2005; Garrido et al., 2008; Cooper et al., 2013). Four presentation blocks were created, one for each domain (speech and non-speech) and dimension (spectrum and duration) combination. For speech spectrum, the paradigm started with 8 tokens of [fε] and continued with 100 trains of [fε] and [fa] each, alternating in series of 4–8 identical stimuli. The count of 4–8 was pseudorandom, fulfilling the condition that each count eventually occurred 20 times. The number of presented tokens was 608 for [fε], and 600 for [fa]; summing up to a total of 1208 stimuli in each block. Stimulus onset asynchrony was 1.09 s. Total presentation time per block was 22 min. The blocks for speech duration were created in an identical way, alternating series of short [fε]s and the long [fε:]s. Analogous presentations were made for non-speech spectrum and non-speech duration. Each participant was tested with either the two speech blocks, or the two non-speech blocks. Stimulus domain thus varied between participants and dimension within participants, with the order of durational and spectral presentation counterbalanced.

### Participants and Procedure

A total of 32 adult volunteers participated in the experiment. They were monolingually-raised native speakers of Czech, ages 18–28 years (mean age 24 years, 19 women, 13 men). They did not have any history of neurological or hearing disorders and reported to be right-handed.

Participants were tested in a quiet room at the Faculty of Medicine in Hradec Králové. Prior to the experiment, they filled in a demographic background questionnaire and signed an informed consent form. Half of the participants was randomly assigned to the speech condition and the other half to the non-speech condition. Within each condition, a participant received two blocks, one presenting changes in stimulus duration and the other with changes in stimulus spectral quality; the order of the blocks was counterbalanced across participants. Between the two blocks, there was a 5-min break. During auditory stimulation, participants watched a muted movie with Czech subtitles. Participants were instructed to focus on the movie and ignore the sounds. The experiment followed the standards for research with humans and was approved by the ethics committee of the Faculty of Medicine in Hradec Králové.

### Electroencephalography and ERP Processing

The EEG was recorded from thirty one Ag/AgCl electrodes Fp1, Fp2, F7, F3, Fz, F4, F8, CP4, C3, Cz, C4, TP8, FT7, P3, Pz, P4, FC3, FC4, FT8, M1, M2, OPz, AFz, P7, P8, T7, T8, CPz, FCz, TP7, CP3 referenced to an electrode placed on the nose. The EEG was recorded at a 3000-Hz sampling rate with a bandwidth of 0.3–100Hz (DEYMED Diagnostic s.r.o., Czechia). After band-pass filtering 0.2–40 Hz using EEGLab (Delorme and Makeig, 2004), the data were down-sampled to 300 Hz and epoched with MATLAB release 2020a (MathWorks, United States). The epoch started 100 ms before and ended 800 ms after the onset of the vowel or the onset of the complex tone; mean voltage of the prestimulus part (from −100 to 0 ms) was subtracted from every epoch.

**FIGURE 1:**
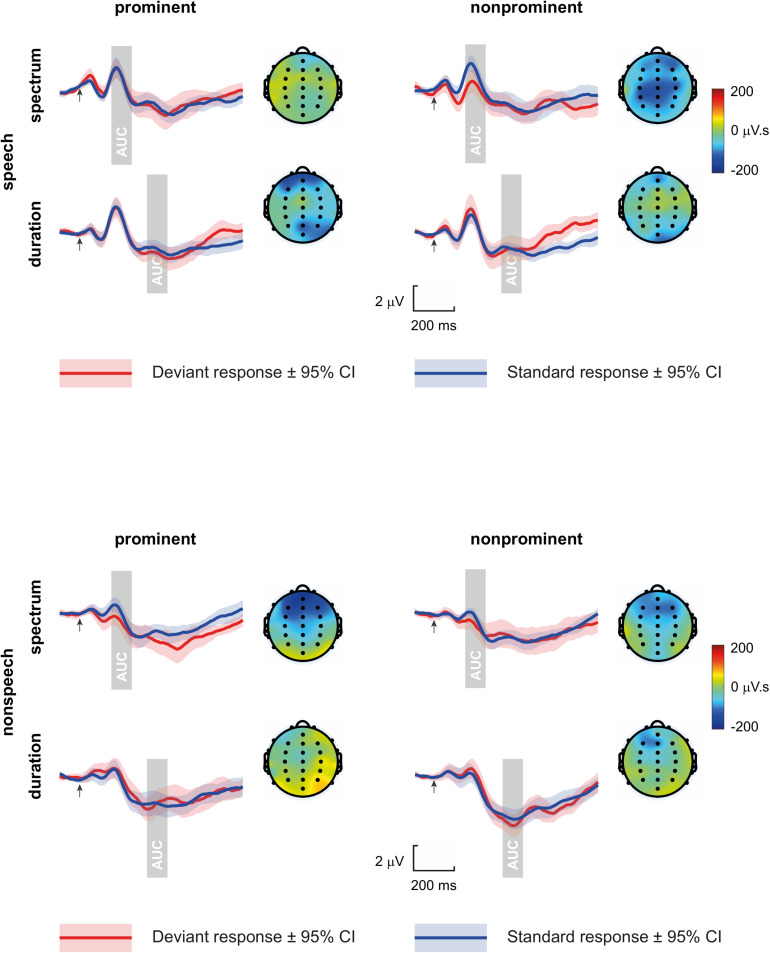
Standard and deviant grand-average waveforms at central channels (averaged across C3, Cz, and C4), and the MMN topographies (displaying the area under curve, AUC, measured in the shaded time windows from deviant-standard differences), per Domain, Dimension, and Deviant type (arrows mark tones/vowels onset).

Deviant waveforms were derived from every first stimulus in the row of 4–8 repeated tokens, standard waveforms were derived from the last two stimuli in the row of 4–8 repeated tokens. Standard and deviant grand-average waveforms at central channels and the MMN topographies are shown in Figure 1. The individual ERPs were calculated as an average of epochs with absolute amplitude under 50 μV. The ERPs were additionally digitally filtered off-line by a smoothing Savitzky-Golay filter (first polynomial order, window of 21 samples).

Difference waves were computed by subtracting the averaged standard ERP from the averaged deviant ERP elicited by physically identical stimuli, e.g., the difference waveform for the [a]-deviant was computed by subtracting the [a]-deviant ERP from the [a]-standard ERP. From the difference waves, the MMN was quantified as area under curve in a pre-defined 100-ms window that started 150 ms after change onset. The window of analysis was determined based on previously published results (Näätänen et al., 1997, 2004; Eulitz and Lahiri, 2004; De Jonge and Boersma, 2015) and visual inspection of the curves, and thus has been set 150–250 ms after vowel or tone onset for the spectral condition and 330–430 ms after vowel or tone onset for the durational condition (where the onset of change was determined as the duration of the short vowel/tone, i.e., 180 ms).

### Statistical Analyses

The calculated AUC were analyzed with a linear mixed-effects model (packages lme4, lmerTest in R, Bates et al., 2015; R Core Team, 2016; Kuznetsova et al., 2017). We modeled the main effects and all two- and three-way interactions of Domain (−speech, +non-speech), Dimension (−duration, +spectrum), and Deviant (−prominent, +non-prominent), as well as the main effects of Laterality (2 contrasts: −left +right, −lateral +midline) and Anteriority (2 contrasts: −central +frontal, −central +parietal). The random effects structure modeled a per-participant intercept and slopes for Dimension and Deviant.

## Results

The summary of the modeled fixed effects is presented in Table 2. As indicated by the significant intercept, overall there was a reliable MMN, estimated as −48 ± 15 μV × ms (*p* = 0.003). The two main effects for Anteriority suggest that the MMN was stronger (more negative) at frontal than at central sites, where it in turn was stronger than at parietal sites, thus following the expected frontally-localized distribution of the auditory and linguistic MMN response.

**TABLE 2:** Fixed-effects summary of the model outcomes.

**Predictor**	**Estimate**	**SE**	**df**	***t***	***p***
Intercept	−**47.999**	**15.150**	**31.738**	−**3.168**	**0.003**
Deviant (−prominent +non-prominent)	12.534	27.848	31.802	0.450	0.656
Dimension (−duration +spectrum)	−31.456	26.836	31.281	−1.172	0.250
Domain (−speech +tone)	4.757	30.299	31.738	0.157	0.876
Laterality (−left +right)	8.084	10.354	1057.792	0.781	0.435
Laterality (−lateral +midline)	−19.745	11.956	1057.792	−1.652	0.099
Anteriority (−central +frontal)	−**46.064**	**11.956**	**1057.792**	−**3.853**	**<0.001**
Anteriority (−central +parietal)	**30.782**	**11.956**	**1057.792**	**2.575**	**0.010**
Deviant × Dimension	17.550	17.138	1068.149	1.024	0.306
Deviant × Domain	−18.349	55.695	31.802	−0.329	0.744
Dimension × Domain	−38.804	53.672	31.281	−0.723	0.475
Deviant × Dimension × Domain	−**189.978**	**34.275**	**1068.149**	−**5.543**	**<0.001**

*Rows marked in bold indicate the effects with *p* < 0.05.*

**FIGURE 2:**
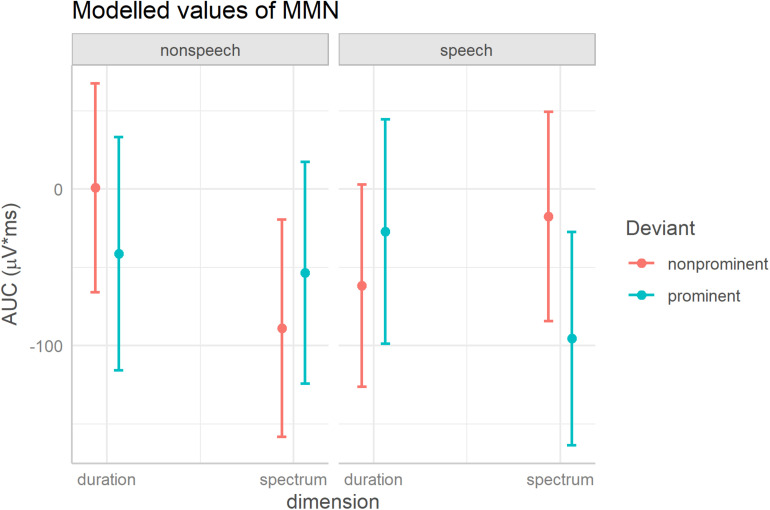
Unpacking the significant three-way interaction of Deviant, Dimension, and Domain. The figure shows model-estimated means and 95% confidence intervals for the MMN elicited by acoustically prominent and non-prominent deviants on each dimension, separately in speech and non-speech stimuli.

Regarding the predictors relevant for our research questions, there was a three-way interaction of Deviant, Dimension, and Domain^2^. To unpack the triple interaction, Figure 2 visualizes the estimated means and confidence intervals [modeled using the R package ggeffects, Lüdecke (2018)]. Pairwise comparisons of the two deviant types on each dimension and in each domain reveal that an asymmetry between the two deviants was found in speech for the spectral contrast: [fa] elicited a stronger MMN than [fε] {[fa] mean = −95 μV × ms, CI = (−164; −27), [fε] mean = −17 μV × ms, CI = (−84; 49)}; in all other conditions the MMNs elicited by the two deviant types overlapped (i.e., the 95% CI’s of one deviant contained the mean of the other deviant, which implies that the difference is not significant at alpha 0.05).

## Discussion

The first question addressed by this experiment was whether the neural processing of phonemic vowel quality differs from the neural processing of phonemic vowel length. To that end, we assessed the neural mismatch response (MMN) in adult speakers of Czech listening to changes between [fε] and [fa] and to changes between [fε] and [fε:] syllables, where both types of change represent a phonological vowel contrast. Our statistical analysis failed to detect a main effect of Dimension (or a two-way interaction of Dimension and Domain). A planned comparison of the MMN elicited by vowel quality (mean = −56 μV × ms, CI = [−111, −2]) and the MMN elicited by vowel length (mean = −44 μV × ms, CI = [−99, 11]) suggests a large overlap across the two types of vowel change, lending support to the conclusion that vowel length and vowel quality changes evoke comparable neural response in Czech adult listeners. Our results for vowels are thus different than the MMN patterns observed by Ylinen et al. (2005) for length and quality changes in plosive consonants.

If we consider the spectral and durational difference between the stimuli in just-noticeable difference units (JND), the Euclidean distance between the first three formants of the [a] and [ε] stimuli is equal to 5.1 JND, whereas the durational difference between the [ε] and [ε:] stimuli equals 12.8 JND [JNDs computed assuming the discrimination threshold of 0.3 bark for vowel formants, Kewley-Port (2001) and a 5 ms discrimination threshold to the reference value of 90 ms for vowel duration, Nooteboom and Doodeman (1980)]. Even though the JND in duration is more than 2 times greater than the JND in spectrum, the average MMNs elicited by each of the changes were not found to differ. Speculatively, this could be taken as an indication that the contrasts have been processed based on their phonological difference rather than the acoustic distance.

The second aim of the experiment was to test whether the vowel contrasts are processed asymmetrically, and if yes, whether the asymmetries are attributable to the acoustic or the phonological properties of the vowels. To that end, we compared the MMN elicited by changes in vowels to the MMN elicited by identical changes in non-speech stimuli. Regarding the spectral contrast, an acoustically-based approach formulated under the NRV framework (Polka and Bohn, 2003, 2011) predicted a larger MMN in case of vowel change from /ε/ to /a/ than vice versa. When comparing vowels /ε/ and /a/, the latter one is auditorily focal, or perceptually more salient, since its first and second formants are close to each other such that they merge into one prominent frequency band. In contrast, the first two formants of /ε/ are farther apart, resulting in vowel /ε/ assigned to the non-focal, perceptually less prominent, element of the comparison. Thus, under the acoustically-based approach, we expected a larger MMN when /a/ was the deviant, and smaller MMN is expected when /ε/ was the deviant in the present experiment. Concerning the durational difference in vowels, a long vowel, here /ε:/, contains acoustic energy over a longer time interval, and is thus inherently more auditorily prominent than a short vowel of the same quality, here /ε/. Therefore, for the change between /ε/ and /ε:/, the acoustically-based approach predicted greater MMN when the long /ε:/ was the deviant than when the short /ε/ was the deviant. Crucially, if perceptual asymmetries in vowels were acoustically conditioned, the same asymmetries were expected to be observed in the non-speech condition, which compared MMN to the changes between /ε/-like and /a/-like complex tones, as well as between /ε/-like and /ε:/-like complex tones. Alternatively, if any detected asymmetries did not conform to the acoustically-motivated predictions, or were not detectable in the non-speech stimuli, they could be attributable to the linguistic status of the vowels. The specific phonologically-based predictions were formulated in line with the FUL (Lahiri and Reetz, 2002, 2010), and predicted an opposite direction of asymmetry due to the phonological feature specification in vowel height. Since /a/ is specified for feature [LOW] and /ε/ is fully underspecified, greater MMN response was expected when /ε/ served as deviant than vice versa. As for the durational contrast, asymmetry would be caused by feature [LONG], which is specified for /ε:/ but not for /ε/, therefore predicting greater MMN response for the short vowel /ε/ deviant.

The statistical model revealed a significant triple interaction of Deviant, Domain and Dimension. Pairwise comparisons of the MMN across the two directions of change (i.e., the two deviants) within each condition (i.e., for each dimension and each domain) revealed an MMN asymmetry for the spectral contrast in speech. A change from [fε] to [fa] elicited a stronger MMN than a change from [fa] to [fε] (no other asymmetries were detected). On the one hand, this result shows that a change from a non-prominent to a prominent vowel is better detectable than a reverse change, which is in line with the acoustically-motivated predictions within the NRV framework and would favor an acoustically-based explanation for the asymmetry. On the other hand, however, this asymmetry was not detected in the non-speech condition where the stimuli differed in identical acoustic parameters as did the stimuli in the speech condition. Due to its lack in the non-speech condition, we conclude that the asymmetry that we found in the processing of the spectral vowel contrast between /a/ and /ε/ is specific to speech and cannot be entirely acoustically based.

Another factor suggesting that the phonologically-motivated explanation for the present MMN asymmetry is more plausible is the duration of stimulus-onset asynchrony (SOA) in our experimental paradigm. SOA was fixed at 1.09 s, which is relatively long, and therefore was more likely to tap into phonological rather than purely acoustic processing (Werker and Logan, 1985). Johnson (2015) addressed the predictions for perceptual vowel asymmetries made by the acoustic and phonological frameworks and has shown that the pattern of vowel perception asymmetry is modulated by the experimental setting. He explored perceptual asymmetries in vowels via reaction time in two discrimination tasks differing in the inter-stimulus interval (ISI), where short ISI (100 ms) implied lower-level auditory listening conditions and long ISI (700 ms) induced higher-level phonemic listening conditions. The results of Johnson’s experiments indicated that the phonological underspecification model of Lahiri and Reetz (2002, FUL) accurately predicted the direction of vowel perception asymmetry in the phonemic conditions, and that in the auditory listening task this direction was reversed, and instead could be explained by the hypotheses employing acoustic characteristics of sounds. Here, we uncover an asymmetry in the processing of vowel quality but did not to detect it in a comparable non-speech condition, with a same, in Johnson’s terms relatively long, ISI across the two conditions (the ISI being 730 or 910 ms depending on vowel/tone duration). It therefore appears that the asymmetry we detected for a spectral contrast in vowels is likely, at least in part, phonologically based.

However, the present asymmetry with a change from [fε] to [fa] eliciting a stronger MMN than vice versa, is opposite to what FUL would predict. Yet it is still possible that an underspecification account be compatible with such a finding if one considers not only the backness feature (as done in most previous MMN studies testing the FUL theory) or if one sees feature specifications as language specific. The Czech vowels /a/ and /ε/ do not differ only in their featural specification of height as we considered (in line with previous studies on similar vowel contrasts in other languages, e.g., /ae/ vs. /ε/ in Scharinger et al., 2012), but also in their featural specification of place. One could thus argue that it was the (under)specification of vowel place rather than vowel height that caused the present perceptual MMN asymmetry. The feature [FRONT] is likely specified for Czech /ε/ but not necessarily for Czech /a/ because in the vowel system of Czech, /a/ (along with its long counterpart) is the only low vowel does not need to be contrasted by the feature place with another low vowel quality (unlike for the mid front vowel /ε/ which contrasts with the mid back vowel /ε/). The explanation that Czech listeners responded more strongly to a mismatch in the phonological specification of vowel place than to a mismatch in the phonological specification of vowel height would also be partially in line with the results of De Jonge and Boersma (2015) who examined MMN asymmetries in French listeners. Those authors found out that the changes between French front rounded and back vowels evoked greater MMN than did the changes between high and mid-high vowels, which indicates that the horizontal difference (in place) between vowels is more salient than the vertical difference (in height).

It is possible that for the Czech /a/-/ε/ contrast a place mismatch is more relevant than a height mismatch, or, that both are relevant phonologically but in the case of the stimuli used here, the place mismatch overrode the height mismatch. Comparing the F1 and F2 of the vowels used in the present experiment, it can be seen that the relative distance between the first formants of [a] and [ε] is less (namely, 2.07 bark) than the relative distance between the second formants of [a] and [ε] (namely, 4.08 bark). Although phonological specification operates on discretized entities, which means that the raw acoustic distance should not matter for whether or not a phonological category contrast is perceived, MMN amplitude is modulated both by linguistic and acoustic differences between standard and deviant stimuli (e.g., Näätänen et al., 1997; Phillips et al., 2000). Therefore, the apparent prime role of underspecification of vowel *place* (rather than vowel height) might as well be, at least partially, driven by the fact that the change in phonological place between the /a/ and the /ε/ was acoustically almost twice as large as the change in phonological height (i.e., 4.08 bark versus 2.07 bark). All in all, if phonological underspecification is extended to vowel place, the present results are explainable as phonologically conditioned asymmetries.

## Conclusion

Pre-attentive processing of changes in phonemic vowel length and vowel quality by adult Czech speakers was assessed in an ERP experiment. The neural mismatch response (MMN) elicited by a change in vowel length between /ε/ and /ε:/ was comparable to the MMN elicited by a change in vowel quality between /ε/ and /a/, suggesting that both types of phonemic changes are equally salient to Czech speakers. For the vowel quality contrast, a perceptual asymmetry was detected where a larger MMN response was found to a change from /ε/ to /a/ than vice versa. No such asymmetrical pattern was observed in non-speech stimuli differing in the same acoustic parameters as the vowels, which indicated that the vowel asymmetry is more likely attributable to the vowels’ linguistic status, namely phonological feature specification, than (purely) to the vowel acoustics. A stronger MMN for the vowel spectral change was elicited by a switch from /ε/ to /a/ than vice versa, from which we have inferred that for this Czech vowel contrast it is the feature specification for place which is primarily exploited by language users. We argued that it might have been a (language-specific) underspecification in terms of place for /a/ (rather than universal underspecification in terms of height for /ε/, assumed by the FUL, Lahiri and Reetz, 2002, 2010) which caused that listeners more readily detected a change from a FRONT /ε/ to an underspecified /a/ than vice versa.

## Data Availability Statement

The data supporting the conclusions of this article and the associated analysis scripts are available from the OSF website at https://osf.io/2849m/. The raw EEG data will be made available by the authors upon reasonable request.

## Ethics Statement

The studies involving human participants were reviewed and approved by the University Hospital Hradec Králové Ethics Committee. The patients/participants provided their written informed consent to participate in this study.

## Author Contributions

JU, JK, and KC designed and implemented the experiment. JU and ZO performed the data collection. NN, JK, and KC processed and analyzed the data. NN wrote the manuscript with contributions and edits from KC, JK, JU, and ZO. All authors contributed to the article and approved the submitted version.

## Conflict of Interest

The authors declare that the research was conducted in the absence of any commercial or financial relationships that could be construed as a potential conflict of interest.

## Publisher’s Note

All claims expressed in this article are solely those of the authors and do not necessarily represent those of their affiliated organizations, or those of the publisher, the editors and the reviewers. Any product that may be evaluated in this article, or claim that may be made by its manufacturer, is not guaranteed or endorsed by the publisher.

## Footnotes

1 We did not adopt the 220-ms stimulus as a short counterpart of the 360-ms /ε/ because the resulting long/short ratio 1.6 is more typical of the high front Czech vowel pair while for mid-low vowels the ratio is closer to 2 (Paillereau and Chládková, 2019).

2 A reviewer expressed concerns about a potentially low power of our experiment. We therefore simulated the power curves associated with an effect equal to the one we obtained, as well as a smaller effect, using the simr package in R (Green and MacLeod, 2016). For the simulations, we created a new model using the parameters of the initial model and calculated its power for various number of respondents for the effect of three-way interaction of Deviant, Dimension, and Domain. The obtained power curve indicated that to reach power of 80%, even with the smaller effect size (i.e., the lower bound of 95% CI of the mean estimated effect in our study) for the critical three-way interaction, a total of 20 participants (i.e., 10 per group) would be sufficient. From this we conclude that our experiment with 32 participants, i.e., 16 per group, is not underpowered.
